# Major Depressive Syndrome (MDS) and its Association with Time of Residence among Spanish Speaking Au-Pairs Living in Germany

**DOI:** 10.3390/ijerph16234764

**Published:** 2019-11-28

**Authors:** Bernarda Espinoza-Castro, Tobias Weinmann, Rossana Mendoza López, Katja Radon

**Affiliations:** 1 Center for International Health, University Hospital, LMU Munich, 80336 Munich, Germany; katja.radon@med.uni-muenchen.de; 2 Institute and Clinic for Occupational, Social and Environmental Medicine, University Hospital, LMU Munich, 80336 Munich, Germany; Tobias.Weinmann@med.uni-muenchen.de; 3 Center for Translational Research in Oncology, Instituto do Câncer do Estado de São Paulo, 01246000 São Paulo, Brazil; rossana@usp.br

**Keywords:** au-pairs, migrants, time of residence, mental health, major depressive syndrome

## Abstract

The number of au-pairs in Germany is on the rise. In 2017, about 13,500 au-pairs were living in German families, almost half of them originating from non-European Union (EU) countries and many of them from Spanish speaking countries. Knowledge about mental health among au-pairs in Germany is limited. Therefore, the main objective of this study was to assess the prevalence of Major Depressive Syndrome (MDS) and its potential association with time of residence among Spanish speaking au-pairs living in Germany via an exploratory analysis. This study included a sample of 409 Spanish speaking au-pairs living in Germany. We classified the au-pairs into those who lived less than three weeks in Germany (newcomer au-pairs) and those who lived more than three weeks (experienced au-pairs). The participants were recruited by an online survey (Facebook and Instagram) from August 2018 to June 2019. Socio-demographic characteristics, time of residence in Germany and the level of education were assessed. MDS was assessed by the Patient Health Questionnaire depression module (PHQ-9). Poisson regression models were calculated to evaluate the association between time of residence in Germany and prevalence of MDS. Most of the participants were female (91%). Almost half of them came from Colombia (48%) and were in the age range between 22–24 years (40%). Prevalence of MDS was 8% among newcomers and 19% among experienced au-pairs (*p* = 0.002). Differences remained statistically significant after adjustment for potential confounders (age, level of education and time of residence in Germany) (prevalence ratio 2.25; 95% confidence interval: 1.22–4.14). In conclusion, au-pairs may develop mental symptoms during their time abroad. Future prospective studies should aim at identifying potential risk factors and preventive measures.

## 1. Introduction

An au-pair (French for “on mutual terms”) is “a usually young foreign person who cares for children and does domestic work for a family in return for room and board and the opportunity to learn the family’s language” [1]. Being an au-pair is considered an opportunity for a young person to get to know another culture as well as to travel, gain experience and learn a language at low cost.

The number of au-pairs in Germany is on the rise (around 1000/year from 2012 onwards) [2]. In 2017, about 13,500 au-pairs were living in German families, almost half of them originating from non-European Union (EU) countries. Most of the au-pairs from EU countries were from Spain, France, and Italy, while from non-EU countries the largest numbers came from Georgia, Ukraine and Colombia. Accordingly, Colombians were the largest group (514 au-pairs in 2017) among Latin Americans, followed by Mexicans and Brazilians. In total, Spanish speaking au-pairs constitute one of the biggest groups of au-pairs in Germany [3]. 

According to the German Federal Agency of Work, in order to be an au-pair in Germany, young foreigners should be between 18 and 28 years, should have completed secondary school education, should have basic German knowledge, and should have a contract with the hosting family minimum for six months and maximum for 12 months [4]. Usually, au-pairs search for families through agencies, via internet platforms, social networks or personal contacts. Agencies, after an application fee, help au-pairs to search for a potential family, to apply for a visa, and to provide support during their time abroad [4].

Au-pairs live in a structural dependency of the employer/host family [5]. Some are perceived as the cheapest way to hire full-time domestic service, which often leads to poor working conditions such as work overload, overtime, and underpayment [6]. For example, according to the 2018 economic survey of developments and trends in au-pair exchange programs, the main problems that au-pairs living in Germany reported were work overtime and having unclear work instructions [3]. Additionally, preliminary results of a cross-sectional study indicate that 12% of the au-pairs experience violence in the family and 3% are suffering from sexual abuse [7]. 

Also, a French study reported that Latin American au-pairs coming to Europe suffer extra challenges because they usually come from families with a middle or high socio-economic status and high levels of education (university degrees). Hence, they are not used to perform household tasks [6]. At the same time, most of them might experience their first job and first time abroad without their families. Other challenges are the foreign language and conflicts between low and high context culture [8]. According to Würtz et al, all cultures are connected to each other through communication styles. In some places, such as Northern European countries, the communication is direct and explicit (low-context culture) [9]. However, in other cultures, such as Latin Americans, an important part of the communication includes body language and implicit messages (high-context culture) [9]. Hence, Latin American au-pairs come from a high-context culture to the German low-context culture, which is another challenge that many of them not even expect. Finally, there usually is lack of preparatory training to au-pairs before going abroad, especially when au-pairs search their host families for themselves. 

Another factor that influences migrants’ mental health and well-being is the time of residence in the host country. For instance, studies suggest that experienced migrants had worse mental health and well-being than newcomer migrants due to the so-called “healthy migrant effect” [10,11,12]. This theory refers to two phenomena. First, migrants arrive in the host country with relatively good physical and mental health (a requirement for immigration and work permits), especially as healthy individuals are more likely to migrate than the rest of their compatriots. They can better handle leaving their home country, family and friends, and starting a new life often in an unknown country. Often, they even are healthier than local born residents in the host country. Then, according to the second phenomenon, progressively this advantage declines or even disappears over a relatively short period [12,13].

In total, such cultural challenges and sometimes poor working conditions among Spanish speaking au-pairs as well as dependence and inexperience may result in poor mental health, especially symptoms of anxiety or depression [14]. However, specific knowledge about mental health among Spanish speaking au-pairs in Germany is limited. Therefore, the main objective of this study was to assess the prevalence of Major Depressive Syndrome (MDS) and its potential association with time of residence among Spanish speaking au-pairs living in Germany. 

## 2. Materials and Methods 

### 2.1. Participants and Sampling 

Data collection for this study was carried out from August 2018 to June 2019. To be eligible for this study, participants needed to satisfy three inclusion criteria: (1) being an au-pair in Germany; (2) being born in a Spanish speaking country; (3) being aged from 18 to 28 years (age required in Germany to work as an au-pair from non-EU countries). Each participant accepted an informed consent form, which contained information about the study objectives, the methodological procedures, and the declarations on the anonymity and confidentiality principles. At the beginning of the survey, the participants created their own identification code with three letters and three numbers (e.g., AFR987). This kept the participants anonymous and gave them the opportunity to resign from the study. The Ethics Committee of the Medical Faculty at the Ludwig Maximilian University of Munich approved the study protocol (project number 18-139).

### 2.2. Data Collection and Questionnaire Instrument 

A total of 409 Spanish-speaking au-pairs living in Germany participated in this study. We used convenience sampling due to the unavailable sampling frame and a dispersed distribution of Spanish-speaking au-pairs in Germany, which is common among “hard-to-reach populations” such as migrants [15]. Hence, we applied two snowball recruitment methods: First, we contacted 16 au-pairs agencies in Latin America (9 in Colombia, 5 in Mexico and 2 in Argentina), 4 in Spain and 3 in Germany. We sent them invitation emails with the link to the online survey asking them to share these invitations with their au-pair candidates (conventional snowball sampling). Secondly, we created Facebook advertising and set a budget for each click (0.10 €), based on the inclusion criteria of the study population. This advertising was posted every week from Friday to Monday assuming that during the weekends the participation would increase (Facebook snowball sampling). Also, we identified 58 Facebook groups of Spanish-speaking au-pairs living in Germany and we posted the link to the online survey with the study information in these groups. To increase participation, we offered an online shopping voucher worth 5 euros to the participants who answered the entire questionnaire. 

An online questionnaire in Spanish (LimeSurvey^®^) with 21 questions was used to collect the data. The questions were taken from the Spanish short version of the European Working Condition Survey [16], and the Quality of Life and Employment, Labor and Health Conditions First National Survey (ENETS) [17]. With these instruments, we assessed socio-demographic characteristics, the level of education and the current job. We also asked about their current job to ensure that the participants were au-pairs at that moment. Major Depressive Syndrome was evaluated by the Patient Health Questionnaire depression module (PHQ-9) [18]. 

### 2.3. Variable Definition

We used the variable time of residence in Germany as the main exposure, which included two categories: (1) “newcomer au-pairs” (living in Germany less or equal than three weeks) and (2) “experienced au-pairs” (living in Germany more than three weeks). According to the Diagnostic and Statistical Manual of Mental Disorders, 5th Edition (DSM-V), “the diagnosis of MDS requires five or more depressive symptoms (included either a depressed mood or anhedonia) to be present within a 2-week period” [19]. Therefore, three weeks after the arrival to Germany was used as a cut point period. As outcome, we used the PHQ-9 to assess MDS. This tool is a 9-item Likert-type scale, where each item corresponds to the nine depressive symptoms criteria from the DSM-IV [20]. For each symptom, the participants selected whether the symptom had bothered them during the previous two weeks: 0 = “not at all”, 1 = “several days”, 2 = “more than the half of the days”, or 3 = “nearly every day” [21]. Major Depressive Syndrome (MDS) was regarded as present if there were at least five positive responses in the “more than half the days” or “nearly every day” categories and one of these responses included depressed mood (Question 1) or anhedonia (Question 2). Other Depressive Syndrome (ODS) was diagnosed if there were two to four positive responses in the “more than half the days” or “nearly every day” categories and one of these responses included depressed mood (Question 1) or anhedonia (Question 2) [21]. For the sensitivity analysis, all participants with ODS and MDS were included in the category “depressive syndromes”. We considered as potential confounders: sex (male, female), age (in three categories: 18–21, 22–24, 25–28 years), region of origin (in four categories: Spain, Colombia, Mexico and Central America, and South America without Colombia), and high education (in two categories: yes or no, where “yes” means at least one year of university).

### 2.4. Statistical Analysis

SPSS^®^ software version 25.0 was used to analyze the data. The descriptive analyses compared newcomers and experienced Spanish speaking au-pairs living in Germany. Nominal and ordinal variables were described as absolute and relative frequencies. Bivariate analyses with Chi-square test were conducted to assess statistical differences between exposure (time of residence in Germany) and the outcome (MDS). Moreover, Poisson regression models with robust variance estimation were performed using socio-demographic characteristics, level of education and time of residence in Germany as predictors and MDS as outcome. According to Coutinho et al., logistic regression works well for estimating the ratio of probabilities of a rare disease [22]. However, prevalence odds ratios (PORs) become a poor estimator for high prevalence diseases [23]. Therefore, differences between prevalence ratios (PRs) and PORs increase when the prevalence of the disease increase as well [24]. Zochetti et al. suggested the implementation of Poisson regression calculating PRs when the prevalence of disease exceeds ten percent [25]. 

Due to the high prevalence of the outcome (19% MDS among experienced au-pairs), we therefore performed a Poisson regression with robust variance estimation, and calculated prevalence ratios rather than odds ratios (ORs). Robust variance estimations help to adjust the overestimation of the variance and produce adequate confidence intervals [22,24]. 

Crude and adjusted PRs were calculated with 95% confidence intervals (95% CI). Missing values (4.15%) were dropped from the analysis, leaving only complete cases (complete-case analysis). 

## 3. Results

Most of the participants were female (91%) and 40% were between 22–24 years old. Almost half of the participants came from Colombia (48%), followed by Mexico (23%). Regarding the level of education, 78% of the participants were highly educated. More than half of the participants were experienced au-pairs (57%). Gender, age, region of origin and level of education were not statistically significantly associated with the time of residence in Germany (Table 1). 

About 25% of the participants presented depressive syndromes, and 14% had MDS. Experienced au-pairs reported a higher prevalence of MDS (19% vs. 8%; *p* < 0.001) (Table 2) and higher prevalence of depressive syndromes (16% vs. 30%; *p* < 0.001) as compared to newcomer au-pairs (Table 3). The remaining variables were not statistically significantly associated with any depressive syndrome. In the adjusted Poisson regression model, the bivariate results were confirmed: experienced au-pairs had more than two times the prevalence of MDS than the newcomers (prevalence ratio 2.25; 95% confidence interval: 1.22–4.14) (Table 2), and almost two times the prevalence of depressive syndromes (prevalence ratio 1.77; 95% confidence interval: 1.13–2.75) (Table 3).

## 4. Discussion

Our results show a high prevalence of MDS among Spanish-speaking au-pairs living in Germany (14%), especially among experienced au-pairs (19%). Time of residence in Germany was statistically significantly associated with MDS.

The high prevalence of MDS in our study is in line with the prevalence rate of depression (20%) among migrant workers in Europe [26]. This number is considerably higher than the prevalence of major depression in the general European population [27,28]. Also, a study in Germany reported a 14% prevalence of MDS and a 29% prevalence of any depressive disorder associated with acculturation level among Turkish migrants [29]. Furthermore, according to Latin American migrants, a high prevalence of distress was shown in Germany (45%) [30] and in the United States (34%) [31]. O’Connor et al. described stressors which might influence poor psychosocial well-being among Latin American migrants such as current jobs, levels of education, working conditions, time of residence in the host country, acculturation level, social and family support, among others [32]. 

Although no data are available on the prevalence of depression among au-pairs, previous studies identified a high prevalence of MDS and other mental diseases among live-in caregivers. Live-in caregivers are temporal migrants living in a private household and providing child or elderly care and thus to some extend comparable to au-pairs [33]. For example, in Canada, Vahabi et al. reported a 23% prevalence of symptoms of depression and a 43% MDS prevalence among live-in caregivers [34]. Lack of privacy, individuals’ powerlessness to have control over their living-working conditions, and overtime work contributed to higher scores of depression [34]. Also, Carlos and Wilson reported that 67% of live-in caregivers experienced poor physical and mental health mainly due to overload and overtime work, living in their employers’ homes, and separation from their families [35]. Moreover, Spitzer et al. stated that live-in caregivers suffered stress due to lack of social and family support in the host country, disobedience from children, work overload, overtime, lack of permanent residency status, lack of food and privacy, and profound loneliness [36].

Another possible reason causing the decrease of psychosocial well-being among au-pairs might be poor working conditions. Even though the migrant status and the working conditions of au-pairs are clearly defined by the German law, au-pairs from non-EU countries are excluded from certain labor rights that regular employees have [37]. Hence, control from pertinent institutions is difficult mainly because the host family’s home is a closed environment [5]. Hence, in cases of poor working conditions or even violence, it is difficult for au-pairs to change or improve their situation because their living place and residence permits are tied to the host families [5,6]. In addition, living and working in the same place might lead to lose boundaries between working and free time [13].

Moreover, in this study, experienced au-pairs reported a higher prevalence of MDS as compared to newcomer au-pairs (19% vs. 8%). This finding is consistent with studies that expressed concerns about the continuous decline of migrants’ mental health from their arrival in the host country, which is defined as the “healthy immigrant effect”. Robert and Gilkinson concluded that newcomer migrants are significantly less likely than non-migrants to report symptoms of depression, anxiety, and other psychosocial distress, but it is unclear whether this health advantage persists over time [11]. Also, a study in four European countries summarized that, over time, migrants presented poorer physical and mental health than non-migrants due to lack of social networks, poor working conditions and difficulties with the non-native language [17]. Moreover, in Canada, 43% of live-in caregivers believed that their health status had worsened since they arrived to the host country [35]. 

Time of residence can also influence mental health. Migrants’ acculturation process is a long-term source of emotional stress [38,39] due to interpersonal and structural challenges specific to their poor working and living conditions [39]. Therefore, Wu and Schimmele concluded that “time of residence is an important factor in the healthy migrant effect, which appears to be disproportionately concentrated among recent immigrants” [39]. 

A strength of this study was the use of an internet-based sampling method (conventional and Facebook snowball sampling), which increased the geographical scope and the sample size of a vulnerable population on a topic which has rarely been studied before [40]. An additional advantage was the usage of an online survey to reach the Spanish-speaking au-pairs because it minimized data entry errors and facilitated the data analysis. Furthermore, we provided an incentive of online shopping vouchers worth 5 euros. We included material incentives because this approach has been shown to increase participation and fulfilment in online surveys in Germany [41]. This method of using incentives lead to a relatively large sample size of 409 Spanish-speaking au-pairs. 

The application of the Spanish version of internationally standardized questionnaires instruments [16,17,18] permits the comparison with other international studies [42]. Furthermore, the fact that the main author (BE) is a Spanish native speaker allowed us to decrease misunderstanding due to the language and also possible culturally misleading interpretations [43]. Moreover, to the extent of our knowledge, this is the first study to assess the prevalence of MDS and its potential association with time of residence among Spanish speaking au-pairs living in Germany.

On the other hand, the study suffers from some potential limitations. First, selection bias may have occurred due to the usage of a convenience sample. For instance, participants already suffering from mental disorders may have been more likely to participate in this study than healthy participants [44]. Second, it was not possible to calculate the response rate, making it hard to evaluate the representativeness of our study population [45]. Third, we included only Spanish-speaking au-pairs in the study. Hence, it is difficult to know to what extent our results can be transferred to au-pairs from other countries. Fourth, as an exploratory study among Spanish-speaking au-pairs in Germany, we did not include in the survey any questions related to the place of residence/employment or pre-existing mental disorders. Omissions of these variables might be fully explained by the experiences of au-pairs with respect to mental health. Therefore, such variables may be included in future research. Finally, the non-probability sampling design limits our ability to generalize the results of the current data. Future studies may thus be designed in a prospective way and collect information on potential risk factors for depression in au-pairs.

To the extent of our knowledge, the present study is the first exploratory approach to investigate mental health among Spanish-speaking au-pairs living in Germany. This knowledge is important to create intervention strategies to prevent a deterioration of mental health among this vulnerable population. Such strategies should prepare and advise au-pairs and their host families before they start living together to have control throughout the year. Interventions could for example be carried out by the agencies or by governmental entities. Also, according to Vahabi and Wong, these interventions and mental health risks should be spread through social networks and social support organizations to reduce isolation and depression among live-in caregivers among whom au-pairs form a special group [34].

## 5. Conclusions

Au-pairs may develop and suffer from poor mental health during their time in Germany. This knowledge is critical and can be used to inform policy makers as well as to find intervention strategies and adequate counseling before and during the au-pairs program. Future prospective studies should aim at identifying potential risk factors. 

## Figures and Tables

**Table 1:** Descriptive data of 409 Spanish-speaking au-pairs by time of residence in Germany.

Characteristics		Missing	Time of Residence		
			Newcomers (≤3 weeks)	Experienced (>3 weeks)	pX^2^
			N = 176	N = 233	
			*n* (%)	*n* (%)	
**Gender**	**Female**	8	156 (89.1)	211 (90.6)	0.87
	Male		16 (10.9)	18 (9.4)	
**Age (years)**	18–21	3	65 (37.4)	70 (30.2)	0.29
	22–24		63 (36.2)	97 (41.8)	
	25–28		46 (26.4)	65 (28.0)	
**Region of origin**	Spain	8	14 (8.1)	17 (7.4)	0.08
	Colombia		90 (52.3)	103 (45.0)	
	Mexico and Central America		46 (26.7)	57 (24.9)	
	South America (w/o Colombia)		22 (12.8)	52 (22.7)	
**Higher education**	Yes	7	139 (81.8)	179 (77.2)	0.26
	No		31 (18.2)	53 (22.8)	
**Depressive symptoms**	DS -	4	123 (71.1)	131 (56.5)	0.01
	DS +		21 (12.1)	30 (12.9)	
	ODS		15 (8.7)	27 (11.6)	
	MDS		14 (8.1)	44 (19.0)	

DS-: none reported depressive symptoms; DS+: at least one of the required screening symptoms is fulfilled, but the total symptom score is below the threshold diagnosis. ODS: Other Depressive Syndrome: 2–4 reported depressive symptoms and one of the symptoms is depressed mood or anhedonia. MDS: Major Depressive Syndrome: ≥5 reported depressive symptoms and one of the symptoms is depressed mood or anhedonia.

**Table 2:** Prevalence of Major Depressive Syndrome (PHQ-9) and results of crude and adjusted Poisson regression models.

Characteristics		Prevalence	Crude PR	Adjusted PR
		*n* (%)	(95% CI)	(95% CI)
**Gender**	Male	2 (5.9)	1	N/A
	Female	56 (15.4)	2.61 (0.64–10.69)	N/A
**Age (years)**	18–21	21 (15.8)	1	1
	22–24	21 (13.2)	0.87 (0.47–1.60)	0.80 (0.38–1.65)
	25–28	16 (14.4)	0.88 (0.45–1.72)	0.78 (0.40–1.50)
**Region of origin**	Spain	3 (9.7)	1	N/A
	Colombia	26 (13.5)	1.38 (0.41–4.60)	N/A
	Mexico and Central America	15 (14.7)	1.52 (0.44–5.25)	N/A
	South America (w/o Colombia)	13 (17.8)	1.84 (0.52–6.46)	N/A
**Higher education**	No	13 (15.7)	1	1
	Yes	44 (13.9)	0.97 (0.51–1.84)	1.13 (0.55–2.28)
**Time of residence in Germany (weeks)**	Newcomers (≤3)	14 (8.1)	1	1
	Experienced (>3)	44 (19.0)	2.20 (1.20–4.04)	2.25 (1.22–4.14)

PR: Prevalence Ratio; 95% CI: 95% Confidence Interval. Adjusted for age, higher education, time of residence in Germany.

**Table 3:** Prevalence of Depressive Syndromes (PHQ-9) and results of crude and adjusted Poisson regression models.

Characteristics		Prevalence	Crude PR	Adjusted PR
		n (%)	(95% CI)	(95% CI)
**Gender**	Male	7 (20.6)	1	N/A
	Female	92 (25.3)	1.20 (0.552.59)	N/A
**Age (years)**	18–21	33 (24.8)	1	1
	22–24	41 (25.8)	1.06 (0.66–1.70)	0.99 (0.60–1.64)
	25–28	26 (23.4)	0.91 (0.53-1.54)	0.85 (0.48–1.52)
**Region of origin**	Spain	6 (19.4)	1	N/A
	Colombia	47 (24.5)	1.25 (0.53–2.93)	N/A
	Mexico and Central America	21 (28.8)	1.48 (0.60–3.68)	N/A
	South America (w/o Colombia)	23 (22.5)	1.16 (0.47–2.86)	N/A
**Higher education**	No	21 (25.3)	1	1
	Yes	77 (24.4)	0.99 (0.60–1.62)	1.06 (0.61–1.83)
**Time of residence in Germany (weeks)**	Newcomers (≤3)	29 (16.8)	1	1
	Experienced (>3)	71 (30.6)	1.76 (1.13–2.73)	1.77 (1.13–2.75)

PR: Prevalence Ratio; 95% CI: 95% Confidence Interval. Depressive syndromes: all participants with ODS and MDS (PHQ-9: Patient Health Questionnaire). Adjusted for age, higher education, time of residence in Germany.
